# Characterisation and Identification of Individual Intact Goat Muscle Samples (*Capra* sp.) Using a Portable Near-Infrared Spectrometer and Chemometrics

**DOI:** 10.3390/foods11182894

**Published:** 2022-09-18

**Authors:** Louwrens C. Hoffman, Prasheek Ingle, Ankita Hemant Khole, Shuxin Zhang, Zhiyin Yang, Michel Beya, Daniel Bureš, Daniel Cozzolino

**Affiliations:** 1 Queensland Alliance for Agriculture and Food Innovation (QAAFI), Centre for Nutrition and Food Sciences (CNAFS), The University of Queensland, Brisbane, QLD 4072, Australia; 2 School of Agriculture and Food Sciences, The University of Queensland, Brisbane, QLD 4072, Australia; 3 Institute of Animal Science, Přátelství 815, 104 00 Prague, Czech Republic; 4 Department of Food Science, Faculty of Agrobiology, Food and Natural Resources, Czech University of Life Sciences Prague, 165 00 Prague, Czech Republic

**Keywords:** carcass, chemometrics, classification, goat meat, infrared

## Abstract

Adulterated, poor-quality, and unsafe foods, including meat, are still major issues for both the food industry and consumers, which have driven efforts to find alternative technologies to detect these challenges. This study evaluated the use of a portable near-infrared (NIR) instrument, combined with chemometrics, to identify and classify individual-intact fresh goat muscle samples. Fresh goat carcasses (n = 35; 19 to 21.7 Kg LW) from different animals (age, breeds, sex) were used and separated into different commercial cuts. Thus, the *longissimus thoracis et lumborum*, *biceps femoris*, *semimembranosus*, *semitendinosus*, *supraspinatus*, and *infraspinatus* muscles were removed and scanned (900–1600 nm) using a portable NIR instrument. Differences in the NIR spectra of the muscles were observed at wavelengths of around 976 nm, 1180 nm, and 1430 nm, associated with water and fat content (e.g., intramuscular fat). The classification of individual muscle samples was achieved by linear discriminant analysis (LDA) with acceptable accuracies (68–94%) using the second-derivative NIR spectra. The results indicated that NIR spectroscopy could be used to identify individual goat muscles.

## 1. Introduction

Meat identification and authentication is one of the applications for which near-infrared (NIR) spectroscopy is considered a valuable tool, as reported by different authors [1,2,3,4,5,6,7]. The utilisation of NIR spectroscopy has been reported by different researchers to have great success in identifying and differentiating between different meat species (e.g., beef, pork, lamb, and chicken) as well as authenticating different homogenized meat muscle samples from the same or different animal species [1,2,3,4,5,6,7,8]. The detection of adulterated, unauthentic, poor-quality, and unsafe meats is still a major task for the meat and food industries [9]. The meat industry as well as consumers have driven efforts to introduce innovative and reliable detection techniques that can ensure the authenticity, quality, and safety of both meat and meat products along the supply and value chains [3,5,10,11].

It has been recognised that the so-called classical analytical techniques are expensive, laborious, time-consuming, and not appropriate to the modern challenges facing the food and meat industries. Therefore, the demand to guarantee the authenticity and safety of both meat and meat products has increased the interest in developing rapid analytical techniques in food and meat industries [2,3,4,5]. Among these rapid techniques, vibrational spectroscopic techniques, such as NIR, mid-infrared (MIR), and Raman spectroscopies, are useful for the determination of meat quality and authenticity because of their intrinsic characteristics (e.g., rapid, reliable, non-destructive, green, relatively inexpensive) [2,3,4,5].

Although NIR spectroscopy has been applied to different commercial and exotic meats (e.g., beef, lamb, pork, chicken, kangaroo, game, etc.) [12,13,14], not many reports were found that evaluated the use of this technique to analyse goat meat samples. Only one study has been reported that assessed the ability of NIR spectroscopy to characterise and authenticate the composition of goat meat samples [15]. The authors of this study evaluated the use of NIR spectroscopy to estimate protein, moisture, connective tissue, ash, and fat contents in two goat muscles, *Longissimus thoracis* (LT) and *L. lumborum* (LL), with great success (coefficient of determination > 0.70) [15].

Although the focus has been on the adulteration of meat using cheaper alternative species, few studies have evaluated the adulteration of expensive fresh meat cuts with cheaper cuts in the same animal species [16]. Typically, the more expensive cuts in a carcass differ in quality and composition from the inferior cuts or muscles. It is therefore of value to the industry to be able to distinguish between different muscles in a mixture of meat products (e.g., high- vs low-value muscle or commercial cuts), thereby providing proof of provenance and quality; a fillet steak sold as a high-value product due to its inherent quality characteristics is indeed derived from the *Psoas major* muscle and not from some inferior muscle.

Thus, the aim of this study was to evaluate the use of a portable near-infrared (NIR) instrument combined with linear discriminant analysis (LDA) to identify, as well as classify, individual and intact goat muscle samples.

## 2. Materials and Methods

### 2.1. Samples

Fresh goat carcasses (n = 35) from different breeds and sexes (male, female), production systems (including commercial farms), and two different experiments were analysed after being slaughtered in a commercial abattoir in Queensland (Australia). The samples were obtained from two different experiments, where in experiment 1, both male and female goat animals were slaughtered, while in experiment 2, only male goats were analysed. The breeds used in these studies were Boer, Boer crosses, and Australian rangeland goats. The goat carcasses were weighed after 24 h (range of 6 to 28 Kg cold carcass weight) and cut in different commercial cuts (e.g., back leg, chump, flap, loin, rack, shoulder), as described by other authors [17]. In this study, the carcasses were weighed, whereafter the muscles in each commercial cut were anatomically dissected. In total, six muscles were dissected and collected for each of the goat carcasses, namely *longissimus thoracis et lumborum* (LTL), *biceps femoris* (BF), *semimembranosus* (SM), *semitendinosus* (ST), *supraspinatus* (SS), and *infraspinatus* (IS). The total number of muscle samples collected and scanned was 210 (35 goats × 6 muscles each).

### 2.2. Near-Infrared Spectroscopy

The NIR spectra of the individual goat muscle samples were collected using a portable NIR instrument (Micro-NIR 1700. Viavi, Milpitas, CA, USA) operating in the wavelength range of 950–1600 nm (10 nm wavelength resolution). The spectra collection and instrument set-up were controlled using the proprietary software provided by the instrument manufacturer (Viavi Solutions, 2015, Milipitas, CA, USA). The spectral data acquisition settings were set at a 50 ms integration time and an average of 50 scans (MicroNIR Prov 3.1, Viavi, Milpitas, CA, USA). For every 10 samples, a reference spectrum was collected using Spectralon^®^. Each muscle was scanned in triplicate, and the average of these spectra was used in further chemometric analysis.

### 2.3. Chemometrics and Data Analysis

The NIR data were exported into The Unscrambler (version X, CAMO, Norway) for data analysis and pre-processing. The NIR spectra were pre-processed using the Savitzky–Golay second derivative (21 smoothing points and second polynomial order) prior to spectra interpretation and chemometric analysis [18]. In this study, principal component analysis (PCA) and linear discriminant analysis (LDA) were used to analyse and classify the muscle samples according to their origin (e.g., type of muscle or breed). The LDA models were developed using the second-derivative NIR spectra and the muscle types as input variables. Models were developed using full cross-validation (leave one out) [19,20]. In addition, the Kennard–Stone approach was used to select samples to be allocated into a calibration and validation set. The ability of the LDA models to classify samples was evaluated using the percentage of correct (%CC) and incorrect (%IC) classifications using the validation set [19,20].

## 3. Results and Discussion

### 3.1. Spectra Interpretation

**Figure 1:**
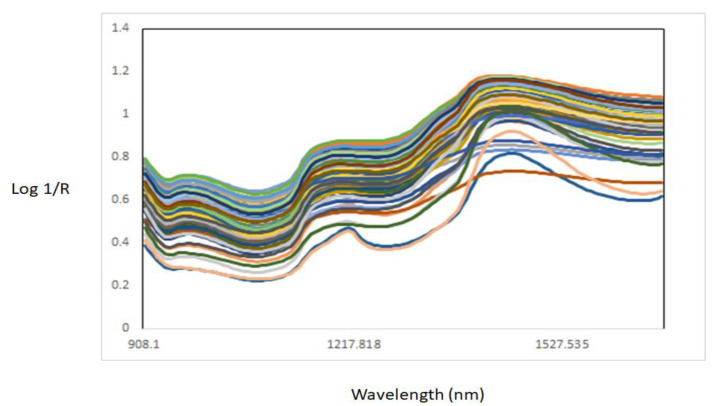
Near-infrared raw spectra of all different intact goat muscle samples analysed.

**Figure 2:**
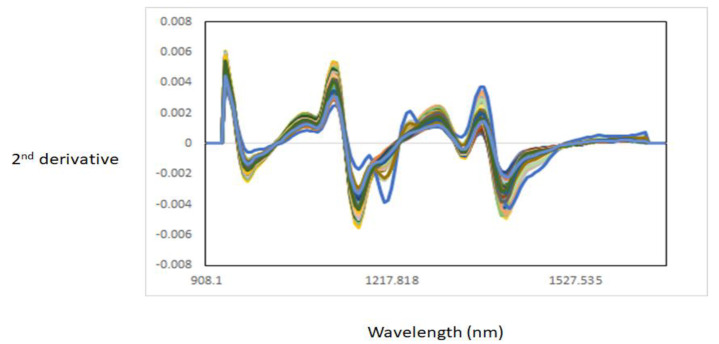
Near-infrared second-derivative spectra of all different intact goat muscle samples analysed.

**Figure 3:**
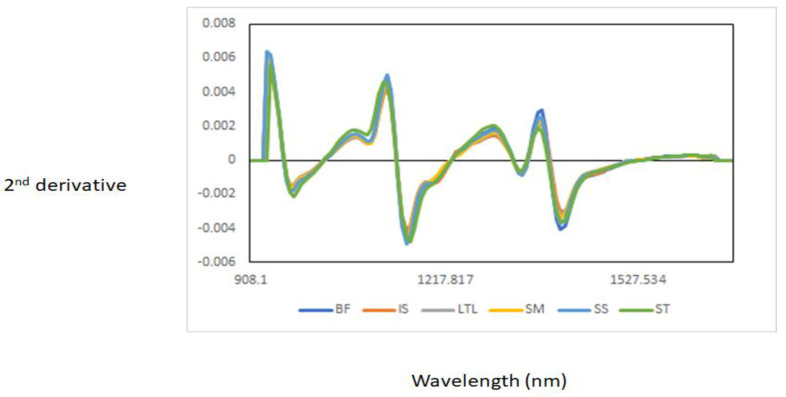
Near-infrared second-derivative average spectra of each of the intact goat muscle samples analysed.

Figure 1 shows the NIR raw spectra of all muscle samples analysed. The raw NIR spectra of the muscles showed three main bands around 976 nm, 1176 nm, and 1428 nm. These bands were associated with third (976 nm) and second (1428 nm) overtones stretching of the O-H bond of water [12,21], while the band around 1176 nm might be associated with the C-H stretching second overtone, associated either with intramuscular fat or lipid content [22,23,24]. An effect of scatter can be observed in the NIR raw spectra of the muscle samples, mainly due to the presence of water. Therefore, the second derivative was used to improve the interpretation of the NIR spectra of the muscle samples analysed (Figure 2). In addition, the average of the second derivative of the NIR spectra of each of the individual muscle samples analysed is also reported in Figure 3. The NIR absorbances throughout the wavelength range of the individual muscle samples analysed overlapped where main throughs (bands) were observed at 976 nm, 1167 nm, 1341 nm, and 1420 nm. A possible explanation for this overlapping might be related to the similarities in the anatomical location, as well as similar functionality of some of the muscles analysed [14,22]. For example, both ST and SS tended to differentiate from the other muscles around 976 nm (water content) and 1167 nm [12,21]. In addition to the differences between ST and SS, BF tended to differentiate from the other muscles at 1416 nm (water content). A change in the NIR spectra could also be observed around 1200 nm, which is associated with lipids and proteins, in muscles such as ST, SS, and IS. Other authors have also reported that differences between muscles (e.g., in chicken) can be observed in absorbances around 980 nm related to the O-H second overtone (water), at 1202 nm related to the C-H second overtone (lipids), and at 1456 nm related to the O-H first overtone (water) [22,23,24]. The band around 970 nm is related to the third overtone stretching of an O-H bond associated with water content [12], while the band around 1143 nm corresponds to the second overtone C-H stretching bonds associated with intramuscular fat and lipids [22]. It is known that the proximate chemical composition of meat is influenced by the sex of the animal, where male animals typically have lower fat and higher moisture content than females [14,25]. Considering that muscles from different goat ages and sex groups were utilized in this study, we can infer that some of the differences observed in the NIR spectra can be associated with the intrinsic differences in intramuscular fat, lipids, and moisture content between animals (age and sex) and muscles (anatomical position and functionality). It has also been observed that some of the muscles overlapped around 1392 nm, associated with the second overtone C-H stretching bond that is related to the lipid content of the samples [22]. Within an animal, muscles are known to differ in their chemical composition, including their moisture and intramuscular fat content [25].

### 3.2. Principal Component Analysis

**Figure 4:**
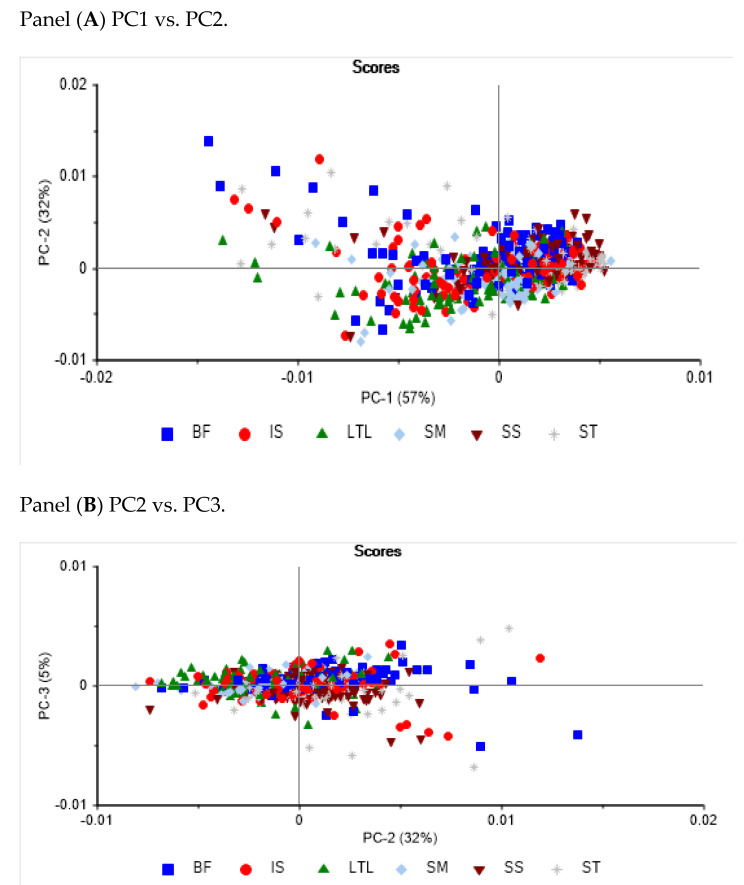

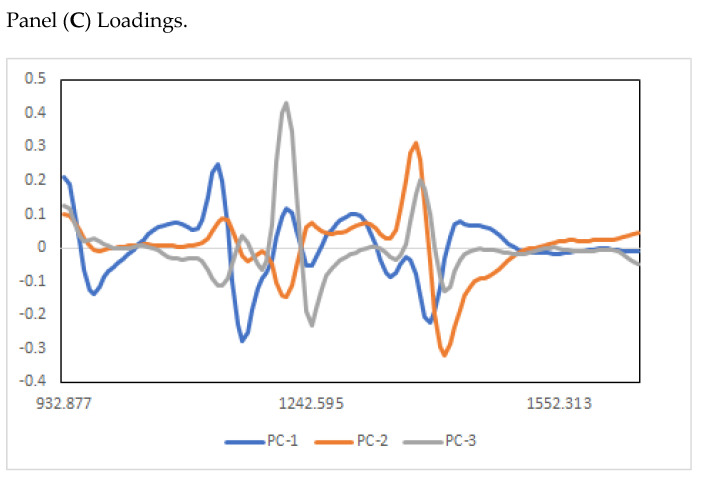
Principal component scores plot (panel (**A**,**B**)) and loadings (panel (**C**)) of intact goat muscles analysed using near-infrared reflectance spectroscopy.

Figure 4 shows the PCA score plot and loadings derived from the second-derivative NIR spectra of the intact goat muscle samples analysed. The PCA analysis showed that 94% of the variance in the NIR spectra of the individual muscle samples is explained by the first three principal components (PC1 57%, PC2 32%, and PC3 5%). Although it is not clear from the figures, similar muscle samples tend to cluster together. This trend can also be observed when PC2 vs PC3 are plotted. Muscles such as SM tend to form a tight cluster, while BF and LTL are scattered along the different PCs. Overall, it is difficult to observe a clear separation between the muscle samples when all the samples are analysed together. The highest loadings in PC1 explained the separation between samples and were observed around 976 nm (O-H), 1180 nm (C-H), and 1428 nm (O-H), associated with water content. The highest loadings in both PC2 and PC3 were similar to those observed in PC1, although some shifts in the wavelength were noticeable. The highest loadings in PC3 were observed at 1112 nm, 1180 nm, 1242 nm, and 1397 nm; both bands at 1242 nm and 1397 nm were associated with fat or lipid content [22].

### 3.3. Classification

The classification results using LDA based on the second-derivative NIR spectra of the individual muscle samples are reported in Table 1. The LDA confusion matrix showed that muscle samples were correctly classified in the range of 63% to 94%, depending on the type of muscle. The poor classification rates were observed for LTL (63%), ST (74%), and SM (71%). For the LTL, 13 samples were misclassified, while 10 and 8 were misclassified for ST and SM, respectively. On the other hand, good to very good classification rates were obtained for BF (82%), SS (94%), and IS (85%), respectively. For BF, six samples were misclassified, while for SS and IS, there were only two and five samples misclassified, respectively. These differences might be attributed to the anatomical and physiological differences among muscles and can also be explained by differences in fibre orientation, muscle chemical composition, physiology, anatomical function, and texture [22,25]. Although the mean second derivative of the NIR spectra appears relatively similar for the different muscle samples analysed, the spectral properties were different, allowing for the discrimination between different muscles.

We also attempted to discriminate muscles according to genotype (e.g., Boer buck, Boer cross, and Australian rangeland). When all muscle samples were analysed together, a classification rate ranging between 52 and 58% was achieved. Thus, comparisons between Boer buck and Australian rangeland, Boer cross, and Australian rangeland, as well as Boer cross and Boer buck, were made separately. Muscle samples were classified correctly with an 80% rate when Boer buck and Australian rangeland were compared. For the other two groups, although an improvement in the classification rate (correct classification around 70%) was achieved, the muscles belonging to the Boar cross were not correctly classified. This might be explained by the fact that Boer buck and cross goats are more genetically similar compared with the Australian rangeland animals. The results of this study indicated that NIR spectroscopy was able to identify the origin of the muscles using intact samples (thus, there is no need for homogenization). These results indicate that NIR use can also be extended to other species and muscles as a high-throughput tool to identify the origin of the meat.

## 4. Conclusions

This study reported the use of a portable NIR spectrometer combined with chemometrics to characterise and identify different goat muscle samples. Differences in the NIR spectra of the muscles were observed around 970 nm, 1242 nm, 1397 nm, and 1428 nm associated with water and fat content (e.g., IMF). The classification of individual muscle samples showed that samples could be classified with accuracies ranging from 68% to 94% using the second-derivative NIR spectra. Muscles that are in the same anatomical location, such as the IS and SS, were correctly classified by NIR spectroscopy. Overall, the results of this study indicated that NIR spectroscopy could be used to characterise and identify different intact goat muscle samples. In future, we can expect an improvement in the NIR models by incorporating samples from other commercial and production conditions, as well as different genetics. The findings of this research might be extended to other species and types of muscles produced and sold within a commercial facility with the several advantages NIR provides, such as the low cost and the fact that this technique it is non-destructive.

## Figures and Tables

**Table 1:** Linear discriminant analysis confusion matrix for the classification of individual goat muscle samples analysed intact by near-infrared reflectance spectroscopy. Results correspond to the validation. In bold is the correct number of samples classified.

	LTL	BF	ST	SM	SS	IS
LTL	**22**	1	0	6	1	5
BF	0	**29**	0	3	2	1
ST	0	1	**26**	1	2	5
SM	1	6	1	**25**	1	1
SS	0	0	0	0	**33**	2
IS	0	0	0	2	3	**30**

LTL: longissimus thoracis et lumborum, BF: biceps femoris, SM: semimembranosus, ST: semitendinosus, SS: supraspinatus, IS: infraspinatus muscles.

## Data Availability

Not applicable.
